# Complete Genome Characterization of a Novel Infectious Bursal Disease Virus Strain Isolated from a Chicken Farm in China

**DOI:** 10.1128/MRA.00632-19

**Published:** 2019-11-27

**Authors:** Guanlong Xu, Jie Li, Qingchun Shen, Lidan Hou, Yingju Xia, Qiuchen Li, Shijie Xie, Shijing Sun, Jiabo Ding, Yaqing Mao, Yuming Qin

**Affiliations:** a National Reference Laboratory for Animal Brucellosis, China Institute of Veterinary Drug Control, Beijing, China; b Department of Population Medicine and Diagnostic Sciences, College of Veterinary Medicine, Cornell University, Ithaca, New York, USA; Portland State University

## Abstract

Infectious bursal disease (IBD) is a highly infectious disease in chicken, and vaccination is the best way to prevent outbreak of infectious bursal disease virus (IBDV). In this study, we isolated a variant IBDV strain from a chicken farm with vaccinated chickens. The full genome of this IBDV strain was determined and analyzed.

## ANNOUNCEMENT

Infectious bursal disease (IBD) is a highly infectious disease that poses a large economic loss to the chicken industry. Infectious bursal disease virus (IBDV) infects mainly the bursa of Fabricius and the lymphocytes, resulting in immunosuppression and subsequent secondary bacterial infection (1).

IBDV is a segmented, double-stranded RNA virus that belongs to the genus Avibirnavirus of the family Birnavirideae (2). The IBDV genome contains two segments of double-stranded RNA, segment A (3,200 nucleotides [nt]) and segment B (2,800 nt). Segment A contains two open reading frames (ORFs); one ORF encodes a polyprotein that is cleaved by proteolysis to produce VP2, VP3, and VP4. VP2 is the major protective antigen for IBDV (3). Segment B encodes only RNA-dependent RNA polymerase (VP1), which is responsible for viral genome replication (4).

In December 2018, an IBDV strain (S18) was isolated from a 25-day-old diseased chicken from a farm in Shandong, China. To characterize the virus, the full genome of the isolate was determined. Briefly, total viral RNA was extracted from the allantois fluid of a chicken embryo infected by strain S18 and subjected to one-step reverse transcription-PCR (RT-PCR) (5), and two pairs of primers were used to amplify the full segment of segments A and B (Seg A-F, GGATACGATCGGTCTGACCCCGGG, and Seg A-R, GGGGACCCGCGAACGGATCCAATTTGG; Seg B-F, GGATACGATGGGTCTGACCCTCTGG, and Seg B-R, GGGGGCCCCCGCAGGCGAAGGCCGG). The resulting PCR products of segments A and B were sequenced by Sanger sequencing by the TsingKe Biological Technology Company (Beijing, China). All of the gene products were sequenced twice to get the final consensus sequence; the final genome was assembled using the SeqMan application in the Lasergene software package (version 7.0) with default parameters.

**FIG 1:**
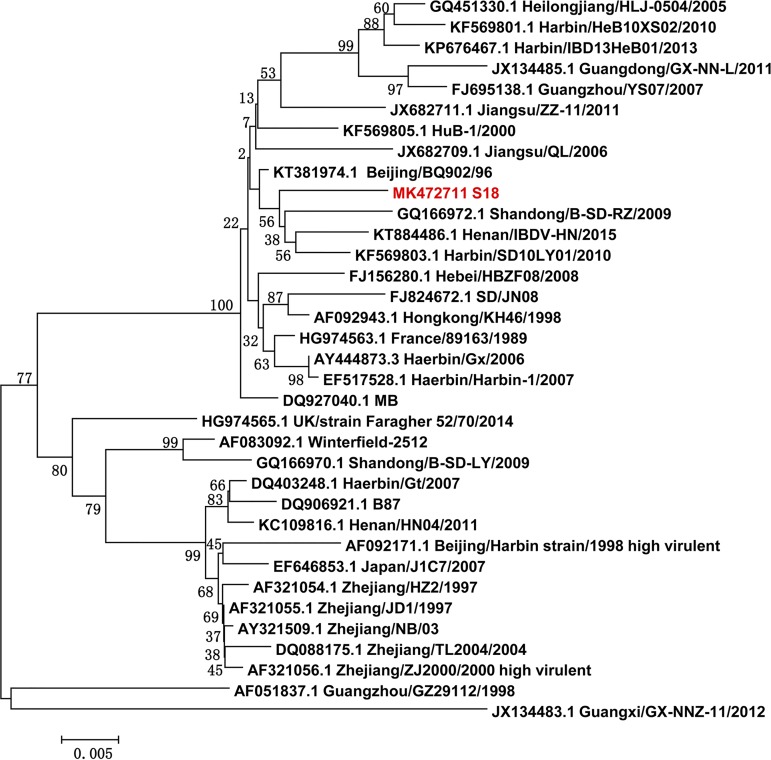
Phylogenetic trees of the VP2 gene of IBDV viruses. Phylogenetic trees were generated by using the neighbor-joining method and bootstrapped with 1,000 replicates using MEGA6 software (version 6.05). Phylogenetic trees were based on the comparison of nucleotide sequences of the IBDV S18 and the reference IBDV sequences from GenBank. IBDV S18 is highlighted in red. Bar, distance unit between sequence pairs.

Sequence analysis showed that segments A and B of S18 contained 3,260 and 2,827 nucleotides, respectively, which showed high similarity (98 to 99%) with the HLJ-7 strain (GenBank accession number GQ452269) isolated from China (Lasergene version 7.0, used with default parameters). Segment B encodes a VP1 polymerase protein that is relatively conserved. The VP1 amino acid sequence of S18 showed 99% identity with that of the reported IBDV strains in China, e.g., the HLJ-7 and Gx strains (6). Segment A encodes VP5 and a polyprotein (cleaved to produce VP2, VP4, and VP3); VP2 is the major protective antigen for IBDV, and it also plays an important role in the virulence of IBDV. Virulence marker amino acids (222A, 242I, 253Q, 256I, and 299S) were observed in the VP2 protein of S18 isolates (7), suggesting that the S18 isolate is a virulent strain of IBDV. A phylogenetic tree of VP2 genes was generated based on the nucleotide sequences of IBDV S18 and the reference IBDV sequences from GenBank, using Molecular Evolutionary Genetics Analysis version 6.05 (MEGA6). The results indicated that S18 showed a distinct relationship with the IBDV vaccine strains used in China, e.g., B87, MB, and W2512 (Fig. 1). The S18 VP2 shared 95 to 98% amino acid identity with those of the vaccine strains; 15 amino acid substitutions were observed in the S18 VP2 protein compared to that of the B87 strain.

In conclusion, we report here the full genome sequence of a virulent IBDV strain isolated from a chicken farm, and the data indicate that a new vaccine matching the IBDV strains circulating in China is warranted to prevent IBD in chickens.

### Data availability.

The complete genome sequence reported here was deposited in NCBI GenBank under the accession numbers MK472711 and MK472712.
